# e-Mental Health Program Usage Patterns in Randomized Controlled Trials and in the General Public to Inform External Validity Considerations: Sample Groupings Using Cluster Analyses

**DOI:** 10.2196/18348

**Published:** 2021-03-11

**Authors:** Samineh Sanatkar, Peter Baldwin, Kit Huckvale, Helen Christensen, Samuel Harvey

**Affiliations:** 1 Black Dog Institute The University of New South Wales Sydney Randwick Australia; 2 School of Psychiatry The University of New South Wales Sydney Randwick Australia

**Keywords:** e-mental health, engagement patterns, external validity, randomized controlled trial, community sample

## Abstract

**Background:**

Randomized controlled trials (RCTs) with vigorous study designs are vital for determining the efficacy of treatments. Despite the high internal validity attributed to RCTs, external validity concerns limit the generalizability of results to the general population. Bias can be introduced, for example, when study participants who self-select into a trial are more motivated to comply with study conditions than are other individuals. These external validity considerations extend to e-mental health (eMH) research, especially when eMH tools are designed for public access and provide minimal or no supervision.

**Objective:**

Clustering techniques were employed to identify engagement profiles of RCT participants and community users of a self-guided eMH program. This exploratory approach inspected actual, not theorized, RCT participant and community user engagement patterns. Both samples had access to the eMH program over the same time period and received identical usage recommendations on the eMH program website. The aim of this study is to help gauge expectations of similarities and differences in usage behaviors of an eMH tool across evaluation and naturalistic contexts.

**Methods:**

Australian adults signed up to myCompass, a self-guided online treatment program created to reduce mild to moderate symptoms of negative emotions. They did so either by being part of an RCT onboarding (160/231, 69.6% female) or by accessing the program freely on the internet (5563/8391, 66.30% female) between October 2011 and October 2012. During registration, RCT participants and community users provided basic demographic information. Usage metrics (number of logins, trackings, and learning activities) were recorded by the system.

**Results:**

Samples at sign-up differed significantly in age (*P*=.003), with community users being on average 3 years older (mean 41.78, SD 13.64) than RCT participants (mean 38.79, SD 10.73). Furthermore, frequency of program use was higher for RCT participants on all usage metrics compared to community users through the first 49 days after registration (all *P* values <.001). Two-step cluster analyses revealed 3 user groups in the RCT sample (Nonstarters, 10-Timers, and 30+-Timers) and 2 user groups in the community samples (2-Timers and 20-Timers). Groups seemed comparable in patterns of use but differed in magnitude, with RCT participant usage groups showing more frequent engagement than community usage groups. Only the high-usage group among RCT participants approached myCompass usage recommendations.

**Conclusions:**

Findings suggested that external validity concerns of RCT designs may arise with regards to the predicted magnitude of eMH program use rather than overall usage styles. Following up RCT nonstarters may help provide unique insights into why individuals choose not to engage with an eMH program despite generally being willing to participate in an eMH evaluation study. Overestimating frequency of engagement with eMH tools may have theoretical implications and potentially impact economic considerations for plans to disseminate these tools to the general public.

## Introduction

Well-designed randomized controlled trials (RCTs) are widely seen as the gold standard for determining treatment efficacy as random assignment to either a treatment or control group allows for the isolation of the treatment effect from both known and unknown confounding factors [1]. Although the importance of RCTs in establishing internal validity is undisputed, researchers have pointed out the external validity concerns of RCTs [2]. These concerns relate to participant selection, attention, retention, researcher contact, and specifics and frequency of data collection—all of which can limit the generalizability of findings to the general public.

Such external validity considerations may be even more justified when considering those RCTs that evaluate e-mental health (eMH) programs, which are designed to deliver effective, scalable mental health care in the community [3-6]. For example, RCTs of eMH programs often selectively recruit from online communities [7] and provide a level of direction for adhering to usage recommendations (eg, reminders) that users in the general public will not encounter, particularly in self-guided eMH programs. These top-down practices affecting user attrition have been described as “push factors” [8]. RCT participants may also be particularly motivated to follow the research protocol and have researcher contact, which likely differs from how the general public experience an eMH program. Furthermore, deterministic approaches to evaluating eMH program effectiveness do not necessarily reflect the ever-changing eMH landscape [5]. As Sieverink and colleagues [9] pointed out, RCTs lack the ability to inform about processes (ie, how behavior evolves between the pre-, post-, and follow-up assessments) or which program components (or combination thereof) contribute to improvements in mental health outcomes. It is therefore pivotal for eMH interventions to show that the treatment effects initially shown in RCTs can translate into real-world benefits in practicable ways.

Despite the need for eMH programs to be applicable to real-world conditions, information on the external validity of eMH treatment outcomes is not widely available. In a recent systematic review of digital interventions addressing comorbid depressive symptoms and substance use, only 1 of the 6 studies examined reported on the comparability of the sample used to the wider population [10]. Similarly, in a review paper of mobile apps promoting physical activity, Blackman and colleagues [11] found that all mobile health intervention studies considered (N=20) reported on treatment effectiveness, but only 4 of these studies reported on how representative the study sample was for the target population.

This problem extends to eMH program design. A considerable body of research examining the effects of different eMH design features on behavioral changes has not addressed the question of how applicable these findings are once the programs are disseminated [3]. Although at least some studies address limitations to the generalization of eMH trial findings, studies examining how or if study protocols influence eMH engagement behaviors are rare. Arguably, whether or not a study protocol influences participant behavior constitutes another important factor in establishing the external validity of eMH findings [4]. One comparison between RCT and real-world uptake was undertaken with moodgym, an eMH program aimed at reducing anxiety and depression. It showed that public registrants were less likely than RCT participants to complete the recommended number of treatment modules [12]. Interestingly, however, symptom reductions over time were comparable between both RCT and community user groups, raising the question of whether a reduced protocol may yield similar benefits.

This short paper attempts to deepen the discussion on the influence of the RCT environment on eMH engagement behavior by presenting engagement patterns of RCT participants and community users of an eMH tool, called myCompass*,* side by side. Our aim is to explore whether patterns of program engagement differ between RCT participants who receive usage recommendations as part of being involved in an evaluation study versus users in the general community who receive the same usage recommendations only on the myCompass homepage. The goal of this paper is to examine engagement rather than outcomes; therefore, we are presenting cluster analysis findings that help visualize behavioral patterns rather than quantify differences in health and well-being.

## Methods

### Program Description

The present analyses are based on the first version of myCompass, an eMH program that was available to all Australians between 2011 and 2018. myCompass is hosted and run by the Black Dog Institute, and funded by the Australian Department of Health. Version 1 of this self-guided program offered online mental health resources and activities to address mild to moderate symptoms of depression, anxiety, and stress. Core functionalities of myCompass were the daily tracking and learning activities components. The tracking function allowed users to track up to 3 moods, behaviors, or cognitions (eg, sadness, alcohol consumption, worry) in real time. Users indicated their current states on an interval scale from low (0) to high (10). Another main function of myCompass was learning activities. Learning activities were a set of 14 modules which aimed at aiding beneficial behaviors such as goal setting, sleep quality, or managing fear and anxiety. Each module was split into 2 to 3 sessions to promote skill-building exercises over the course of several days and took about 10 to 15 minutes to complete online.

### Samples

**Figure 1:**
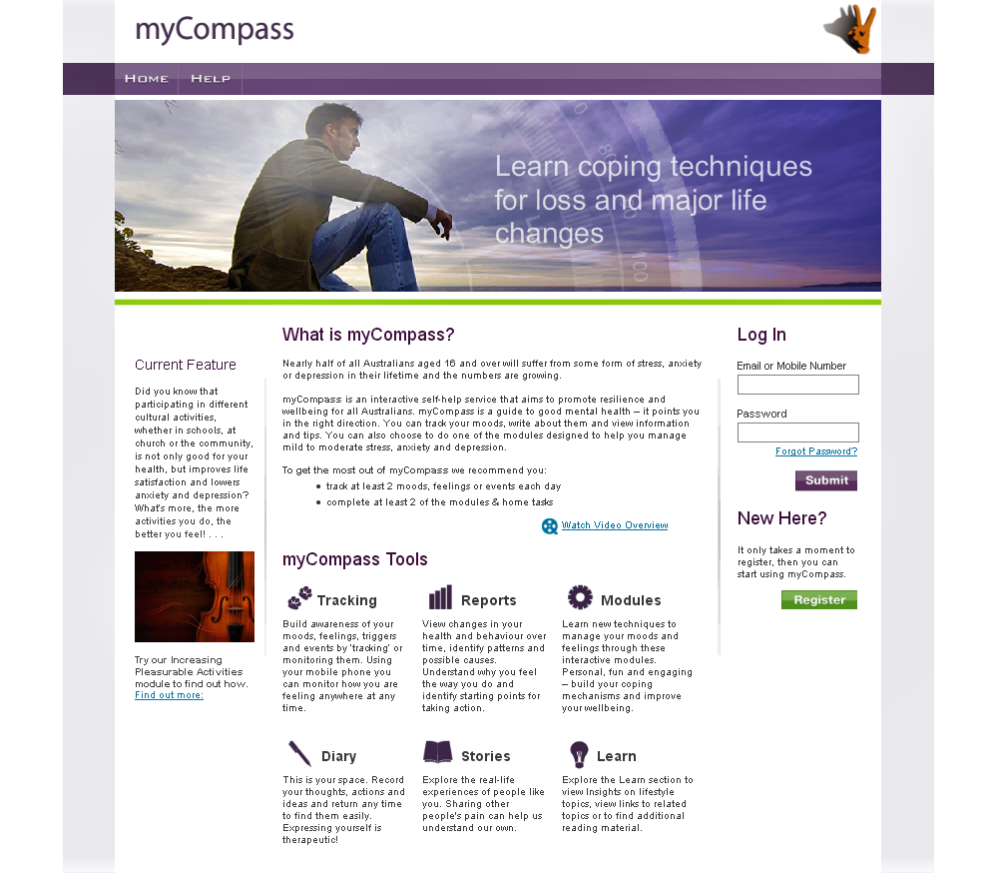
Screenshot of myCompass homepage with usage recommendations.

Our study considered engagement data from 2 distinct samples: (1) an RCT participant sample and (2) a naturalistic community sample of general public users who freely adopted myCompass. All participants and users registered to version 1.0 of myCompass between October 2011 and October 2012. Trial participants and community users freely registered either to the research study or directly to the myCompass website. Trial recruitment took place through multiple avenues, such as social media posts on Facebook and announcements on the Black Dog Institute volunteer research register [13], while the myCompass website could be found spontaneously through internet search engines. RCT participants and general public users received the same usage recommendations on the myCompass home page of completing at least 2 modules and using the tracking function once a day (see Figure 1). All users, independent of whether or not they were part of the research study, set the frequency of program use reminders in line with their own preferences on the myCompass website. However, only research participants were able contact research staff.

The RCT sample comprised 231 participants allocated to the eMH treatment group for the initial evaluation study of myCompass [13]. Exclusion criteria for entering the study were being younger than 18 or older than 75 years, not possessing an internet-enabled mobile phone, not having access to a computer with internet and email, and showing either minimal or severe symptoms of depression and anxiety as determined by symptom scores on the Depression Anxiety and Stress Scale [14].

The community sample comprised 8391 adults who registered for myCompass on their own accord. Community members needed to provide a valid email address and mobile phone number to verify their willingness to register to the program. As part of the profile setup within the myCompass program, RCT participants and community users alike completed assessments of common mental health symptoms, which formed the basis for tracking recommendations made by the program [13]. If scores indicated severe distress (ie, depression, anxiety, or a stress scores of 8 or higher out of 10) or suicidal ideation, the sign-up process was terminated and individuals were redirected to the Black Dog Institute website with information on how to seek immediate support.

### Measurement of User Engagement

We selected 4 usage metrics to assess user engagement. These were number of logins, number of logged trackings (irrespective of how many items were tracked at any one time), number of learning activities started, and number of learning activities completed.

### Statistical Analysis

All analyses were conducted in SPSS version 25 (IBM Corp). Participants using myCompass as part of the RCT and those in the community sample were initially compared using chi-square and *t* tests. We then conducted 2 two-step cluster analyses to determine distinct usage groups within the RCT participant and community user samples. A two-step cluster analysis allows for the detection of naturalistic (ie, not hypothesis-driven) groupings within a data set by way of examining distances between data points (step 1). Based on these distance calculations, an algorithm (step 2) determines the number of groupings or clusters for each data set. In the current analysis, we selected the log-likelihood distance measure and the Schwarz Bayesian information criterion statistic to determine the most appropriate number of clusters.

## Results

### Demographic Information

Data were available for 8622 users of myCompass. Table 1 shows the basic demographic information and online engagement behaviors of individuals taking part in the myCompass RCT and those signing up to myCompass via the program’s website. Although both groups had similar gender ratios (RCT: 160/231, 69.6% female; general public: 5563/8391, 66.30% female; *P*=.30), community users were on average 3 years older than RCT participants (*P*=.003). Notably, all average access and engagements statistics were higher for RCT participants than for general public users (all *P* values <.001).

**Table 1:** Mean, SD, and between-group statistics on demographic information and usage behavior through 49 days (N=8622).

Variable	RCT^a^ sample (n=231), mean (SD)	Community sample (n=8391), mean (SD)	*F* test	*P* values	d
Age (years)	38.79 (10.73)	41.78 (13.64)	9.07	.003	0.20
Female (%)	1.70 (0.46)	1.66 (0.47)	1.07^b^	.30	N/A
49-day logins (n)	11.26 (15.78)	3.90 (6.91)	229.21	<.001	1.01
49-day trackings (n)	11.26 (10.42)	2.57 (5.95)	343.48	<.001	1.24
49-day mod^c^ started (n)	1.14 (1.62)	0.53 (0.85)	113.81	<.001	0.71
49-day mod completed (n)	1.02 (1.53)	0.12 (0.53)	555.85	<.001	1.57
					

^a^RCT: randomized controlled trial.

^b^Chi-square statistic.

^c^mod: modules.

### Two-Step Cluster Analyses

A number of RCT participants allocated to the myCompass group did not proceed to register to myCompass (73/231, 31.6%) and therefore did not provide any engagement data. To make the RCT group more comparable to the general public sample (where each person registered to myCompass and provided engagement data), we removed these participants from the sample used for the cluster analysis. Table 2 shows the results of the cluster analysis among RCT participants who were allocated to myCompass for the duration of the 7-week intervention period. The cluster analysis yielded 2 distinct user groups among the RCT participants. Results of a multivariate analysis of variance confirmed significant cluster group differences on all 4 usage variables (all *P* values <.001; see Table 2), indicating that the clustering procedure was successful in establishing distinct user groups.

The first and larger user group (114/158, 72.2%) were “10-Timers” who logged into their assigned program around 9 times (mean 8.84, SE 2.01) and used myCompass mainly for tracking (mean 7.77, SE 1.83) rather than completing learning activities (mean 0.23, SE 0.21). The second user group (44/158, 27.8%) were “30+-Timers”, who logged into myCompass every other day (mean 36.20, SE 1.71). When the 30+-Timers logged on, they used the program’s tracking function (mean 34.59, SE 1.56) in addition to starting and completing around three learning activities (modules started: mean 3.36, SE 0.19; modules completed: mean 2.67, SE 0.18) over the course of 7 weeks.

**Table 2:** Cluster analysis groupings of 158 myCompass randomized controlled trial participants’ usage behaviors and multivariate analysis of variance testing of the significant difference between clusters.

Variables	Nonstarters (n=73), mean (SE)	10-Timers (n=114), mean (SE)	30+-Timers (n=44), mean (SE)	*F* test (1, 156)	*P* values
Age (years)	38.79 (10.73)^a^	37.08 (10.71)	41.40 (8.09)	5.82	.02
Female (%)	1.70 (0.46)^a^	1.70 (0.46)	1.66 (0.48)	0.24	.62
Logins (n)	N/A	8.84 (2.01)	36.20 (1.71)	184.53	<.001
Trackings (n)	N/A	7.77 (1.83)	34.59 (1.56)	214.73	<.001
Mod^b^ started (n)	N/A	0.77 (0.22)	3.36 (0.19)	145.22	<.001
Mod completed (n)	N/A	0.23 (0.21)	2.67 (0.18)	156.73	<.001

^a^Demographic information on Nonstarters is included for informational purposes and was not part of the analysis.

^b^mod: modules.

Table 3 presents the results of the cluster analysis in the general public. Similar to findings among RCT participants, the community sample cluster analysis yielded 2 distinct clusters that differed across all usage variables (all *P* values <.001). The vast majority of general public users (7681/8391, 91.54%) were “2-Timers” or individuals who entered myCompass approximately twice (mean 2.25, SE 0.17) and used the tracking function once (mean 1.13, SE 0.14). The average module completion neared zero in this group (mean 0.03, SE 0.02).

The second and considerably smaller group were the “20-Timers” (710/8391, 8.46%) who used myCompass consistently. Members of this group logged in on average 22 times (mean 21.82, SE 0.16) and used the tracking function the majority of the time (mean 18.06, SE 0.14) over a 7-week period. In addition, 20-Timers started about 2 modules (mean 1.98, SE 0.03) and completed 1 (mean 1.07, SE 0.02) during this time.

Graphical representations of the cluster solutions can be found in Figures S1 and S2 in Multimedia Appendix 1.

**Table 3:** Cluster analysis groupings of 8391 myCompass general public users usage behaviors and multivariate analysis of variance testing of the significant difference between clusters.

Variables	2-Timers (n=7681), mean (SE)	20-Timers (n=710), mean (SE)	*F* test (1, 8389)	*P* values
Age (years)	41.70 (13.67)	42.83 (13.33)	4.59	.03
Female (%)	1.66 (0.47)	1.67 (0.47)	0.08	.78
Logins (n)	2.25 (0.17)	21.82 (0.16)	13803.55	<.001
Trackings (n)	1.13 (0.14)	18.06 (0.14)	14057.60	<.001
Modules started (n)	0.40 (0.03)	1.98 (0.03)	3145.51	<.001
Modules completed (n)	0.03 (0.02)	1.07 (0.02)	3670.39	<.001

## Discussion

This paper presents findings on individual usage behaviors for an Australian eMH tool, either as part of an RCT or as an open-access tool freely adopted by the general public. Exploratory findings reveal that the same number of usage groups emerged in both data sets: a large “lower-intensity” usage group and a smaller “higher-intensity” usage group. However, our findings revealed interesting differences between the 2 data sets that warrant consideration. First, the general community group tended to be older than the RCT group and overall used the eMH program significantly less frequently based on all usage metrics. Of further note, a considerable number of individuals registered to myCompass directly and were not part of the research trial. This could be because participation in a research study is more time intensive, as research volunteers are required to complete psychometric measures in addition to using the eMH tool. It is also possible that only a relatively smaller number of individuals were exposed to the research trial recruitment calls, while a greater number of interested individuals were able to discover the myCompass website using internet search engines.

Second, although the cluster analytical findings revealed 2 behavioral groups across both samples, the magnitude of usage was higher in the RCT sample for both usage groups. For example, the low-usage group in the RCT logged in an average of 9 times, while the low-usage group in the general community logged in only about twice on average. The high-usage group in the RCT sample was, again, not only higher in magnitude (about 35 logins on average as opposed to about 20 logins in the general community), but also proportionally bigger than that in the community sample. Specifically, 27.8% (44/158) of RCT users were identified as frequent users (30+-Timers), whereas only 8.46% (710/8391) of general public users were identified as such (20-Timers). Accordingly, the 30+-Timers RCT group completed the recommended 2 or more learning activities and came closest to the tracking recommendations of 49 logged trackings, whereas the 20-Timers community sample group clearly did not meet the tracking or learning activity recommendations. Thus, only the high-usage RCT group could be described as “adhering” to the learning activity recommendations, and no group adhered to the tracking recommendations.

Our findings add weight to Cavanagh’s [4] concerns about external validity in eMH trials, suggesting that inferences about real-world engagement from RCT data indeed should be made with caution. Although sample composition and usage patterns were comparable between the RCT and general community users, generalizing from the RCT sample would have overestimated the magnitude of real-world program engagement. One potential reason for this could be the differing motivation for eMH adoption. In our study, RCT participants seemed to be more motivated to use the eMH program and to use the core functionalities more consistently than were users in the general public. It is possible that, beyond the willingness to participate in mental health research, the aims stated in the Participant Information Statement inadvertently attracted individuals who were interested in the topic of eMH and therefore more motivated to engage with an eMH tool in general and with the activities recommended to them in particular. On the other side of the behavioral spectrum, we uncovered a unique set of individuals in the RCT population who were willing to participate in the RCT but did not proceed to register to the online mental health tool (ie, Nonstarters). These participants were not representative of the community population because they did not encounter the eMH tool at all.

Our study is unique in that we contrasted RCT and general public behavioral patterns for the same eMH program during the same time period, maximizing the comparability of users’ eMH experience while minimizing the influence of historical factors. However, some limitations of our analyses warrant consideration. First, our analytic techniques only allowed for a limited number of engagement variables, but many other variables, such as the number of tracking reminders a user sets, could have provided us with a more detailed picture of eMH engagement behavior. Second, we were only able to study those users who actually engaged with the core functionalities of the program repeatedly; therefore, our findings largely reflect individuals motivated to adopt an eMH program. Third, the data reported in this paper were collected between 2011 and 2012 and certainly would have made a timelier contribution then. Technological advances since this time include improvements in interface design and user experience features, such as chatbots, gamification, and virtual reality [15,16]. However, these relatively more high-tech solutions have yet to be fully integrated into the digital mental health landscape [16]. Thus, we believe that the general knowledge and discussion derived from this analysis, which inspected usage behaviors along common metrics such as logins, module usage, and mood monitoring, still bears relevance today. Fourth, the observed effect may be limited in scope. It is possible that the findings presented in this paper only apply to unguided eMH interventions. Usage patterns may differ for eMH programs that provide therapist assistance, which generally facilitates engagement [17]. Last, we did not examine the significance of differences observed across samples. This short paper focused on eMH engagement rather than outcomes, as our goal for this study was to reignite a discussion of the real-world applicability of eMH engagement data derived from RCT findings.

In summary, our findings suggest that eMH engagement in RCTs likely matches the type of eMH engagement in real-world users, but may overestimate the magnitude of such engagement. This could be an important consideration for eMH researchers, designers, and policy makers, as they implement eMH tools after efficacy is established. We recommend that future eMH trials examine whether participant selection and per protocol instructions affect usage behavior, and if so, that due consideration be given to this in implementation planning. Similarly, those planning a wide-scale rollout of new eMH tools should consider which aspects of the original trial may help in promoting usage and whether similar methods can be used in the real world. Ecological validity in eMH engagement science is also relevant to theoretical investigations of how eMH tools improve individuals’ well-being. Mechanisms of change established in RCTs must be practicable in the real world for eMH to deliver on its promise of effective and scalable mental health care. Ultimately, the goal of improving eMH engagement science is to set realistic expectations of eMH benefits—both health and economic—and understand how to maximize these. The more accurately we can speak to eMH engagement, the more fruitful both eMH science and policy will be going forward.

## Appendix

Multimedia Appendix 1 Supplementary figures.

## Abbreviations

eMH: e-mental health
RCT: randomized controlled trial
