# Supplementary material for: e-Mental Health Program Usage Patterns in Randomized Controlled Trials and in the General Public to Inform External Validity Considerations: Sample Groupings Using Cluster Analyses
Source: J Med Internet Res. 2021 Mar 11;23(3):e18348. doi: 10.2196/18348 (PMC7995072; doi:10.2196/18348)
Supplement: Multimedia Appendix 1 [file jmir_v23i3e18348_app1.docx]

This is a Multimedia Appendix to a full manuscript published in the J Med Internet Res. For full copyright and citation information see http://dx.doi.org/[10.2196/18348](https://doi.org/10.2196/18348)

**Supplementary Figure 1: Cluster model summary, cluster quality, and cluster distribution plots of myCompass randomised controlled trial participants**

**
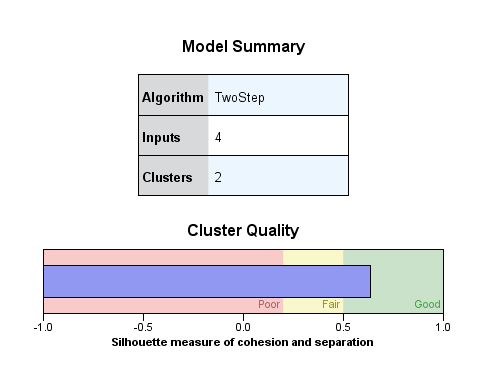
**

**
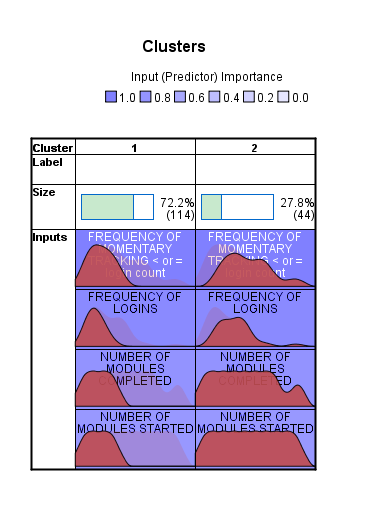
**

30+-Timers

10-Timers

| Tracking |
| --- |
| Logins |
| Modules Completed |
| Modules Started |

**
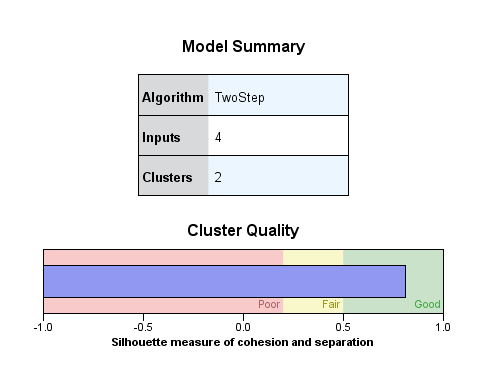
Supplementary Figure 2: Cluster model summary, cluster quality, and cluster distribution plots of myCompass general public users**


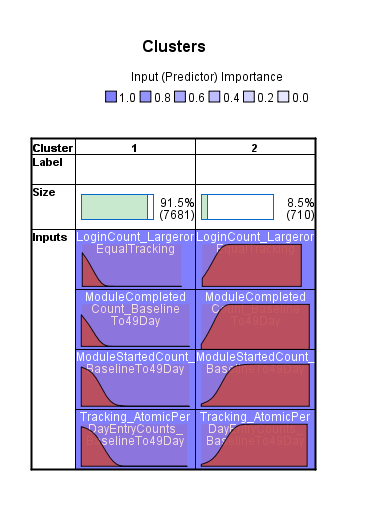


20-Timers

2-Timers

| Logins |
| --- |
| Modules Completed |
| Modules Started |
| Tracking |
